# Virtual simulation scenario for teaching standard precautions: a validation study

**DOI:** 10.1590/0034-7167-2025-0346

**Published:** 2026-10-19

**Authors:** Cristine Maria Pereira Gusmão, Laís do Espirito Santo Lima, Mayra Gonçalves Menegueti, Cristina Mara Zamarioli, Elucir Gir, Renata Karina Reis

**Affiliations:** I Universidade de São Paulo. Ribeirão Preto, São Paulo, Brazil

**Keywords:** Education, Personal Protective Equipment, Simulation Training, Validation Study, Infection Control, Enseñanza, Equipo de Protección Personal, Entrenamiento Simulado, Estudio de Validación, Control de Infecciones

## Abstract

**Objectives:**

to develop and validate a virtual simulation scenario for teaching Standard Precautions and the donning and doffing sequence for personal protective equipment for nursing students and professionals.

**Methods:**

methodological study of technological production. The scenario was developed based on scientific recommendations and simulation guidelines. Experts in simulation and infection prevention and control validated the scenario by completing questionnaires developed by the authors. Data were analyzed using the content validity index and Gwet’s AC1 coefficient, with minimum acceptable values of 0.80 for the CVI and 0.41 for AC1.

**Results:**

the scenario was validated by 11 experts. The final version comprised 20 essential items and showed CVI and Gwet’s AC1 values above the predefined minimum.

**Conclusions:**

the validated scenario shows educational potential and introduces an innovative approach to simulation, helping prepare students and professionals for infection prevention.

## RESUMEN

**Objetivos:**

desarrollar y validar un escenario de simulación virtual para la enseñanza de las precauciones estándar y de la secuencia de colocación y retirada del equipo de protección personal dirigido a estudiantes y profesionales de enfermería.

**Métodos:**

estudio metodológico de producción tecnológica. El escenario se construyó a partir de recomendaciones científicas y directrices de simulación. Especialistas en simulación y control de infecciones asociadas a la atención sanitaria lo validaron mediante cuestionarios elaborados por las autoras. Los datos se analizaron con el índice de validez de contenido y el coeficiente AC1 de Gwet, con valores mínimos aceptables de 0,80 y 0,41, respectivamente.

**Resultados:**

validado por 11 especialistas, el escenario quedó integrado por 20 ítems esenciales y presentó IVC y coeficiente de Gwet superiores al mínimo establecido.

**Conclusiones:**

el escenario validado muestra potencial educativo y carácter innovador, y contribuye a la formación de estudiantes y profesionales para la prevención de infecciones.

## INTRODUCTION

Health care settings pose significant biological risks, and Standard Precautions-such as hand hygiene and the use of personal protective equipment (PPE)-are the primary strategy for ensuring safety for all^(1-3)^. Although recommended by global organizations such as the World Health Organization and the Centers for Disease Control and Prevention (CDC), adherence to Standard Precautions remains insufficient among both professionals and health professions students^(4-6)^.

At the root of the problem are educational gaps, as curricula do not adequately develop essential practical competencies, including infection prevention and control measures. Findings from studies conducted in Brazil and elsewhere, together with challenges observed during the COVID-19 pandemic, reveal shortcomings in the teaching of crucial procedures, such as the recommended sequence for donning and doffing PPE^(6,7)^.

In this context, virtual simulation is an effective teaching and learning strategy because it fosters the development of clinical reasoning and cognitive and psychomotor skills in a safe, realistic environment with immediate feedback, thereby improving performance and self-confidence^(8-10)^. However, its use for teaching Standard Precautions remains limited, and validated scenarios for specific practices are still lacking^(6,11)^.

## OBJECTIVES

To develop and validate a virtual simulation scenario for teaching Standard Precautions and the sequence for donning and doffing personal protective equipment, as an effective teaching tool for nursing education and continuing education.

## METHODS

### Ethical aspects

The study followed national and international ethical guidelines. The institution’s Research Ethics Committee approved the study under opinion No. 6,782,234, issued in April 2024. All participants involved in the validation process received information about the study objectives and provided electronic consent in Research Electronic Data Capture (REDCap^®^), in accordance with recommendations for research conducted in virtual environments.

### Study design, period, and setting

This methodological study^(12)^ of technological production was conducted between December 2023 and August 2024 at a public higher education institution in a non-metropolitan area of São Paulo state, Brazil. The process followed the Revised Standards for Quality Improvement Reporting Excellence (SQUIRE 2.0) checklist for developing and validating a simulation scenario focused on Standard Precautions.

The scenario structure was based on the National League for Nursing/Jeffries Simulation Theory (NLN/JST)^(13)^ and the Healthcare Simulation Standards of Best Practice™ of the International Nursing Association for Clinical Simulation and Learning (INACSL)^(14)^. The technical content was grounded in current evidence and guidelines on Standard Precautions and PPE use^(1,2)^, as illustrated in Figure 1.

**Figure 1:**
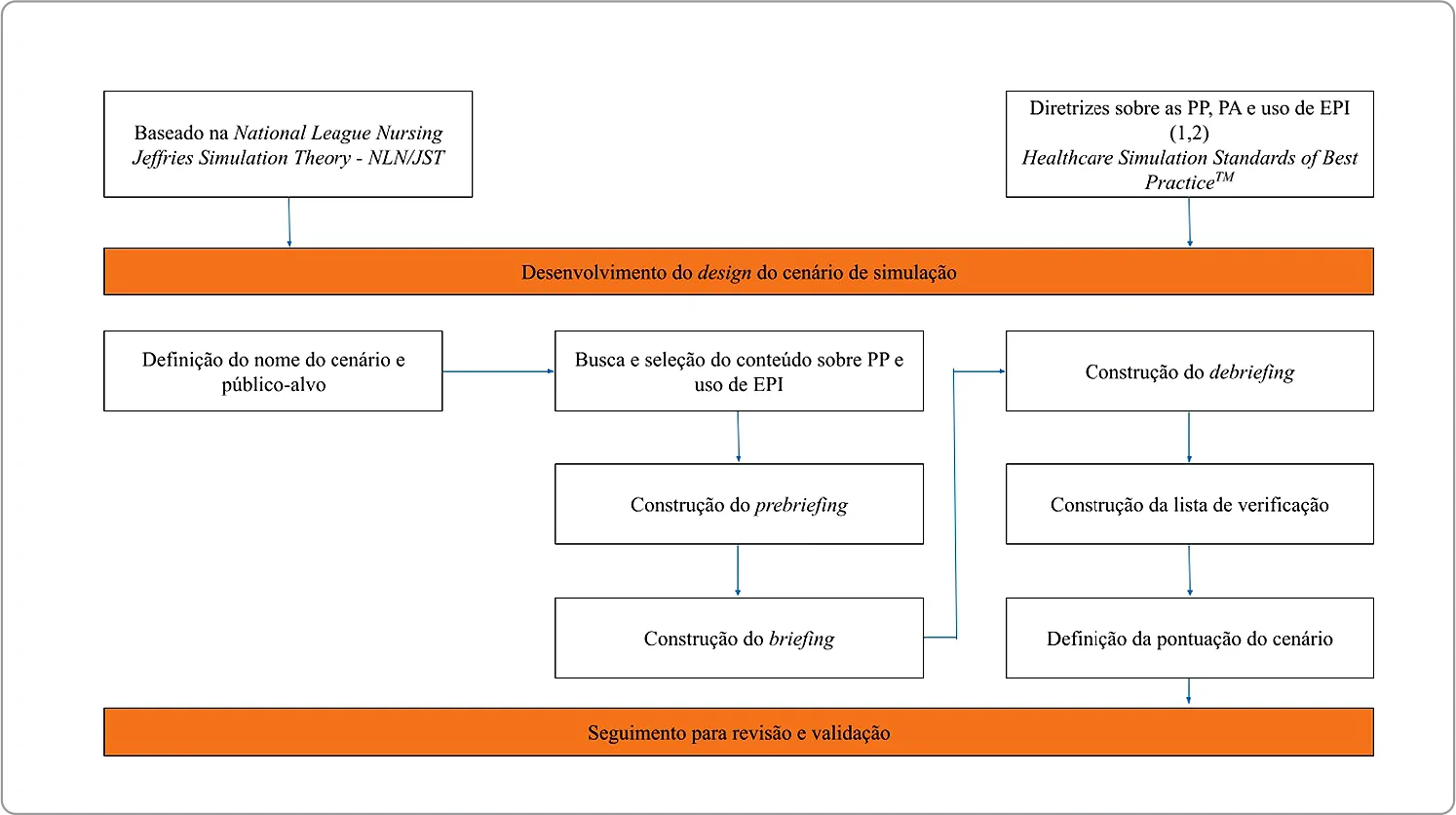
Flowchart of the development of the virtual simulation scenario for teaching Standard Precautions, Ribeirão Preto, São Paulo, Brazil, 2025

*Traduction: Based on the National League for Nursing/Jeffries Simulation Theory (NLN/JST); Guidelines on Standard Precautions, Transmission-Based Precautions, and PPE use (1,2); Healthcare Simulation Standards of Best Practice™; Development of the simulation scenario design; Defining the scenario title and target audience; Searching for and selecting content on Standard Precautions and PPE use; Development of the prebriefing; Development of the briefing; Development of the debriefing; Development of the checklist; Definition of scenario scoring; Proceeding to review and validation*

Face and content validity were assessed by experts in simulation and/or infection prevention and control, based on criteria described in the literature^(15)^.

### Population and inclusion criteria

Experts were eligible if they held an undergraduate degree in a health-related field and met the minimum score required under a scoring system adapted from Fehring^(15)^, according to which participants had to score at least 5 points based on academic credentials, professional experience, and scientific output in the field. The scoring system adopted was as follows: a master’s degree in the study area or topic (3 points); a doctoral degree in the study area or topic (3 points); a graduate certificate in the study area (2 points); a master’s or doctoral degree in another field (1 point); at least 1 year of clinical experience in nursing (1 point); publications in areas such as simulation and/or infection control (2 points); studies conducted in the topic area (2 points); supervision of academic work on the topic (1 point); clinical or teaching experience; and research conducted or authorship of scientific articles on the topic published in journals (1 point).

For recruitment, experts were identified on the Lattes Platform using the “Search CV” feature, with filters for academic degree, subject area, and keywords such as “infection control” and “simulation”. Next, 27 professionals were contacted by email. The message included information about the study and a link to the electronic Informed Consent Form (ICF), allowing recipients to choose whether to participate. After an initial response from five experts, snowball sampling was used, and participants were asked to recommend professionals with expertise in the topic. Thus, 11 experts were included-a number considered sufficient to exceed the minimum of five experts recommended for validation studies^(16)^.

### Data collection

Between May and August 2024, data were collected using the REDCap platform. After providing online consent, 11 experts completed a sociodemographic questionnaire and evaluated the developed scenario.

Scenario validation was carried out using a researcher-developed questionnaire modeled on a study with a similar purpose^(17)^. The instrument included items assessing the material for face validity (clarity and realism)^(16)^ and content validity (appropriateness and relevance)^(16)^, rated on a Likert-type scale^(18)^ ranging from 1 to 5, where 1 corresponded to “Not clear”, “Not appropriate”, and “Not relevant”, and 5 to “Very clear”, “Very appropriate”, and “Very relevant”. It also included an optional open-ended field for participants to suggest refinements to the scenario.

### Data analysis

Data were exported from REDCap^®^ for analysis in R software (version 4.4.1)^(19)^, and participant characteristics were summarized using descriptive statistics. Content validity was assessed using the content validity index (CVI), calculated as the proportion of ratings of 4 or 5 out of the total number of ratings provided by the experts, with ≥ 0.80 adopted as the acceptance criterion^(12)^. Interrater agreement was additionally assessed using Gwet’s AC1 coefficient (first-order agreement coefficient)^(20,21)^. AC1 was selected because, unlike Cohen’s kappa, it is less affected by prevalence-related distortions and marginal homogeneity^(20)^. AC1 values were interpreted as follows: < 0.40 = poor; 0.41 to 0.75 = satisfactory to good; 0.75 to 1.00 = excellent^(22)^. Participants’ suggestions were analyzed and incorporated into the scenario to refine it, based on the literature.

## RESULTS

### Scenario

The scenario comprised 20 items. Its framework specifies the scenario title, target audience, setting, purpose, time frame, modality, difficulty level, scenario scoring, prerequisite competencies, theoretical framework, learning objectives, and expected outcomes. The operational script includes the materials, case description, scenario progression, an action checklist, assessment, and the prebriefing and debriefing stages, as illustrated in Figures 2 and 3.

**Figure 2:**
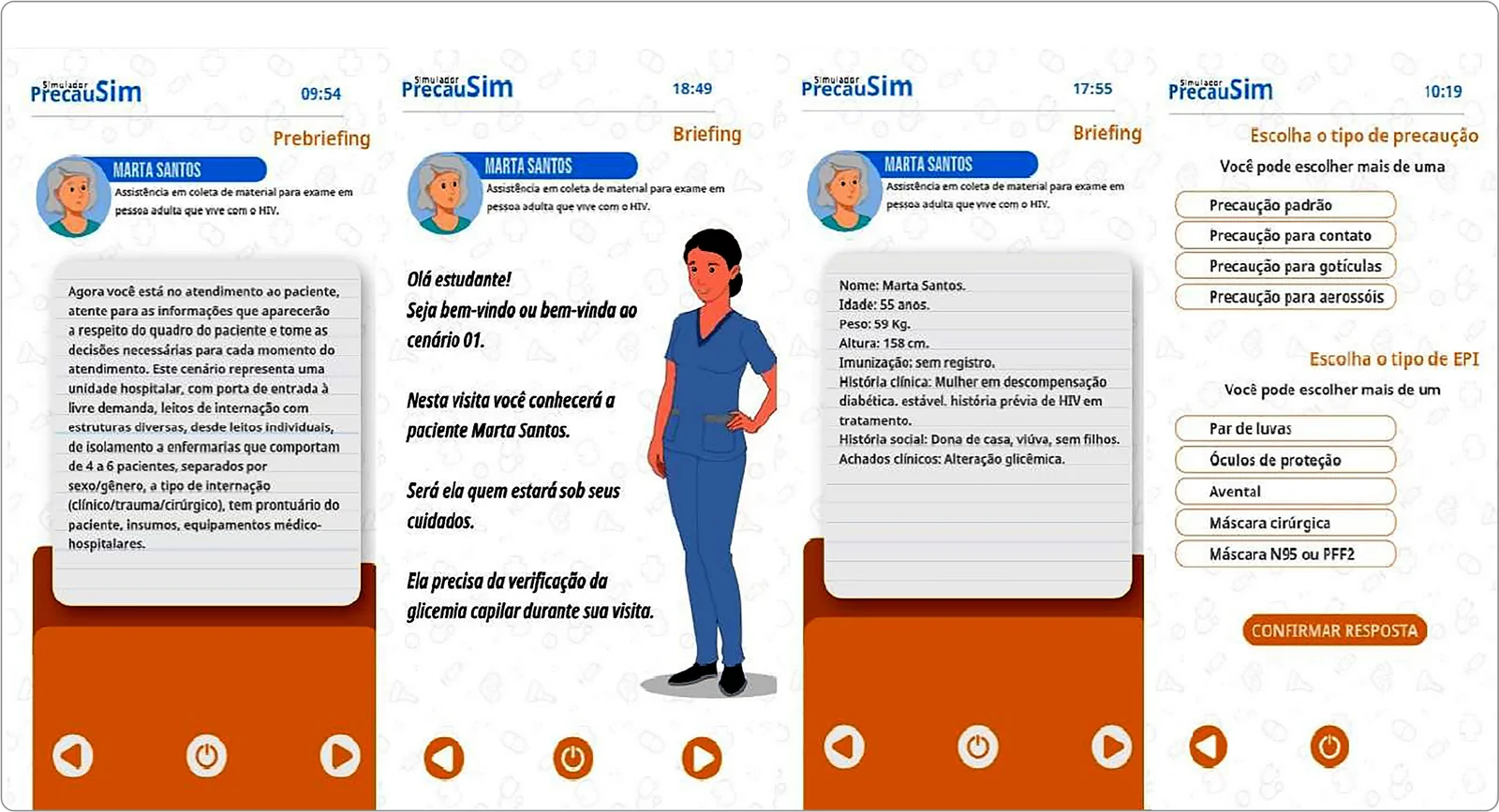
Screenshots from the virtual simulation scenario for teaching Standard Precautions validated by experts (N = 11), Ribeirão Preto, São Paulo, Brazil, 2025

*Traduction:* **Marta Santos** *Assistance with specimen collection for testing in an adult living with HIV;* **Screen 1 - Prebriefing** *You are now caring for the patient. Pay attention to the information displayed about the patient’s condition and make the decisions required at each stage of care. This scenario represents a hospital unit with a walk-in admission area and a range of inpatient accommodations, from private and isolation rooms to wards accommodating 4 to 6 patients, separated by sex/gender and type of admission (medical/trauma/surgical). The patient chart, supplies, and medical equipment are available.;* **Screen 2 - Briefing** *Hello, student!; Welcome to Scenario 01.; During this visit, you will meet patient Marta Santos.; She will be under your care.; She needs capillary blood glucose monitoring during your visit.;* **Screen 3 - Briefing** *Name: Marta Santos; Age: 55 years; Weight: 59 kg; Height: 158 cm; Immunization: no record; Clinical history: Woman with decompensated diabetes. Known HIV infection under treatment; Social history; Homemaker, widowed, no children; Clinical findings: Altered blood glucose level;* **Screen 4 - Choose the type of precaution** *You may choose more than one.; Standard Precautions; Contact Precautions; Droplet Precautions; Airborne Precautions;* **Choose the type of PPE** *; You may choose more than one.; Gloves; Protective eyewear; Gown; Surgical mask; N95 or PFF2 respirator;* **CONFIRM SELECTION**

**Figure 3:**
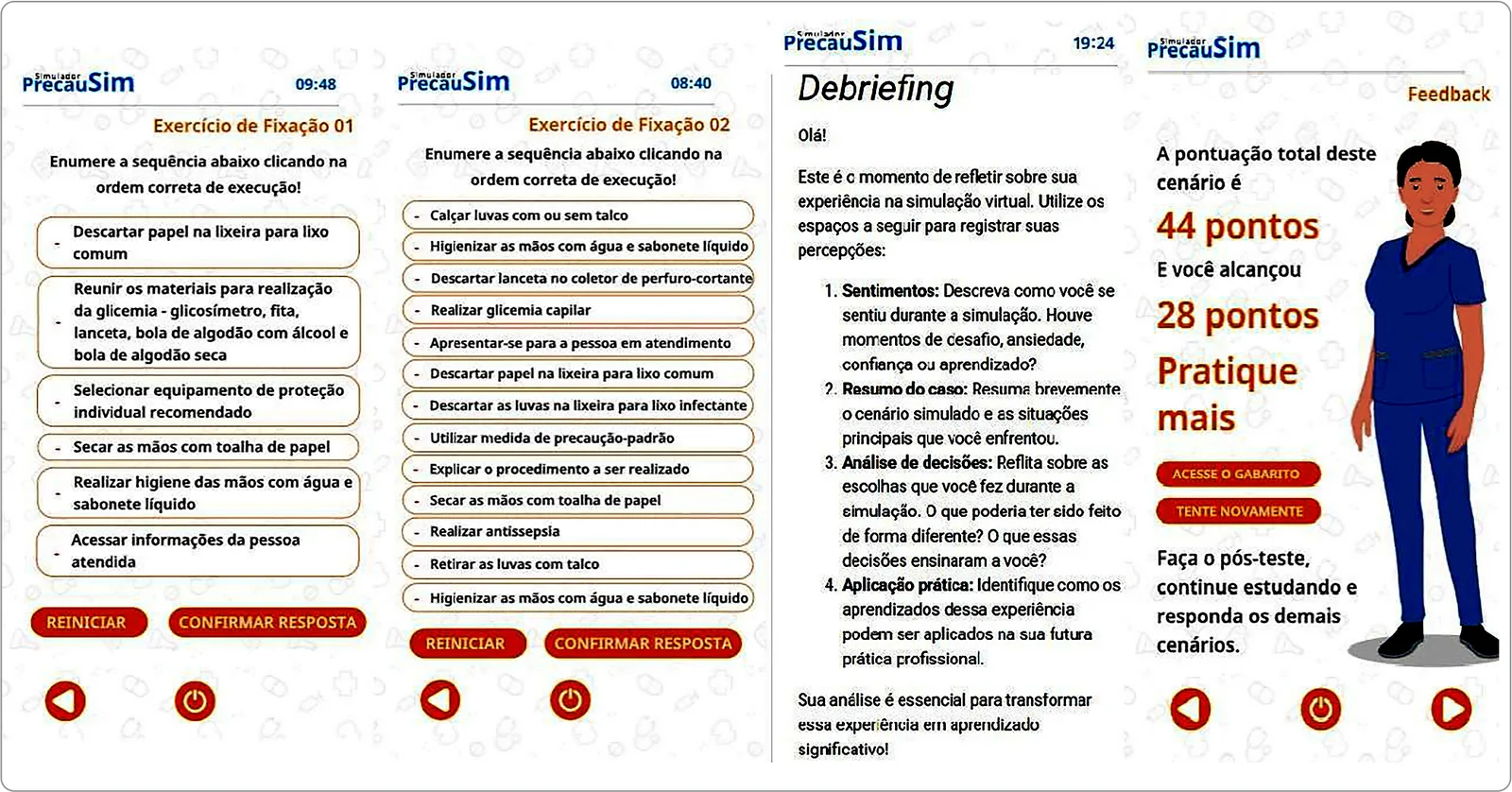
Screenshots from the virtual simulation scenario for teaching Standard Precautions, validated by experts (N = 11), Ribeirão Preto, São Paulo, Brazil, 2025

*Traduction:* **Screen 1 - Practice Exercise 01** *Arrange the sequence below by selecting the steps in the order they should be performed. Discard paper in the regular trash bin Gather the materials needed for blood glucose testing: glucometer, test strip, lancet, alcohol-soaked cotton ball, and dry cotton ball. Select the recommended personal protective equipment*

*Dry your hands with a paper towel. Wash your hands with soap and water. Check the patient’s information* **RESTART**; **SUBMIT ANSWER**; **Screen 2 - Practice Exercise 02** *Arrange the sequence below by selecting the steps in the order they should be performed. Put on powdered or powder-free gloves. Wash your hands with soap and water. Dispose of the lancet in the sharps container. Check capillary blood glucose. Introduce yourself to the patient. Discard paper in the regular trash bin. Discard the gloves in the infectious waste bin. Follow Standard Precautions. Explain the procedure to be performed. Dry your hands with a paper towel. Clean the site with antiseptic. Remove the powdered gloves. Wash your hands with soap and water.* **RESTART**. **SUBMIT ANSWER**. **Screen 3 - Debriefing** *. Hello! This is the time to reflect on your experience in the virtual simulation. Use the spaces below to record your perceptions:* **1. Feelings:** *Describe how you felt during the simulation. Were there moments of challenge, anxiety, confidence, or learning?* **2. Case summary:** *Briefly summarize the simulated scenario and the main situations you encountered.* **3. Decision analysis:** *Reflect on the choices you made during the simulation. What could have been done differently? What did those decisions teach you?* **4. Practical application:** *Identify how the lessons learned from this experience can be applied to your future professional practice. Your reflections are essential to turning this experience into meaningful learning!* **Screen 4 - Feedback** *The total score for this scenario is* **44 points** *You scored* **28 points Practice more VIEW ANSWER KEY TRY AGAIN** *Complete the post-test, keep studying, and complete the remaining scenarios.*

### Validation of the simulation scenario for teaching Standard Precautions

#### Characteristics of the experts

Validation was conducted by a panel of 11 expert nurses, most of whom were women (n = 10; 90.9%), with a mean age of 36.6 years. The experts had a mean of 24.5 years of professional experience. All members of the expert panel held graduate degrees, with six (54.5%) holding doctoral degrees and five (45.5%) holding master’s degrees.

Regarding areas of practice, ten (90.9%) worked in both teaching and clinical care, one (9.1%) had experience with simulation, and one (9.1%) worked in nursing with a focus on infection prevention and control. The experts’ scientific output further underscores their expertise in the topic area: seven (63.3%) had publications on Standard Precautions and simulation, and eight (72.7%) had publications on HAIs.

#### Evidence of face and content validity

In the face and content validity assessment, most scenario items achieved a content validity index (CVI) above 0.80, as shown in Table 1. Items scoring below this threshold were revised based on the experts’ suggestions.

**Table 1:** Distribution of expert ratings for the clarity, appropriateness, and relevance of the simulation scenario for teaching Standard Precautions (N = 11), Ribeirão Preto, São Paulo, Brazil, 2025

Scenario items	Criterion		
	Clarity	Appropriateness	Relevance
	CVI-C^‡^	CVI-A^§^	CVI-R^||^
Scenario title	0,91	1,00	0,91
Target audience	0,91	0,91	1,00
Setting for simulation and debriefing	0,82	0,73	0,73
Purpose	1,00	0,91	1,00
Time frame	0,91	0,91	1,00
Modality	0,91	0,82	0,82
Difficulty level	0,91	1,00	0,91
Scenario scoring	1,00	1,00	1,00
Prerequisite competencies	0,82	0,91	1,00
Theoretical framework	0,91	0,91	0,91
Learning objectives	0,91	0,91	1,00
Expected outcomes	0,73	0,82	1,00
Materials to be selected	0,82	0,73	0,82
Prebriefing	0,91	0,91	1,00
Assessment	0,91	1,00	1,00
Setting characteristics and scenario script	0,91	0,91	0,91
Case description	1,00	0,91	0,91
Scenario progression	0,91	0,91	0,91
Checklist and expected actions	0,82	0,91	0,82
Debriefing	0,91	0,91	0,82

*CVI-C^‡^ - content validity index for clarity; CVI-A^§^ - content validity index for appropriateness; CVI-R^||^ - content validity index for relevance.*

Interrater agreement, assessed using Gwet’s AC1 coefficient, was considered satisfactory. For the specific scenario items, the values were 0.58 for clarity, 0.47 for appropriateness, and 0.50 for relevance. For the general items, the corresponding values were 0.49, 0.42, and 0.53, respectively (Table 2).

**Table 2:** Distribution of expert responses in the agreement assessment of the general and specific items of the simulation scenario for teaching Standard Precautions (N = 11), Ribeirão Preto, São Paulo, Brazil, 2025

	Agreement			
	**Criterion**	AC1^*^	**SD^†^**	**CI^§^**
**General scenario items** Target audience, setting (for simulation and debriefing), purpose, time frame (prebriefing, briefing, and debriefing), modality, scenario scoring, prerequisite competencies, theoretical framework, learning objectives, expected outcomes, prebriefing, assessment, materials to be selected, and debriefing	Clarity	0.49	0.05	(0.378.0.597)
	Appropriateness	0.42	0.06	(0.281.0.546)
	Relevance	0.53	0.06	(0.393.0.659)
			
				
**Specific scenario items** Scenario title, difficulty level, setting characteristics and script, case description, scenario progression, checklist and expected actions	Clarity	0.58	0.06	(0.435.0.725)
	Appropriateness	0.47	0.05	(0.353.0.588)
	Relevance	0.50	0.05	(0.373.0.616)

* *AC1 - Gwet’s AC1 coefficient (first-order agreement coefficient); †SD - standard deviation; §95% CI - 95% confidence interval.*

The scenario for teaching Standard Precautions was refined based on expert feedback. At the request of two experts, the item “Materials to be selected” was revised to “Materials and actions to be selected”.

The items “Expected outcomes” and “Setting for simulation and debriefing” were retained unchanged because no specific suggestions for revision were made. Although “Setting for simulation and debriefing” had a CVI below 0.80, the decision to retain it was based on two factors: the absence of proposed revisions and the nature of the project. Because the scenario was developed for an online platform, the authors concluded that no viable alternative was available for that section.

The general comments provided in the suggestion field did not call for direct textual revisions; instead, they addressed the importance of the material and the proposal’s online format, which participants viewed positively.

The final consolidated version of the scenario, together with the validation data, is available in the supplementary material.

## DISCUSSION

This study developed and validated a virtual simulation scenario for teaching Standard Precautions, with a focus on the steps involved in and the sequence of PPE donning and doffing. The findings provided evidence of face and content validity for the proposed context, with content validity indices above 0.80 and satisfactory agreement among the experts.

In Brazil, the use of simulation-based learning (SBL) in nursing has been increasing, placing greater demands on educators and reinforcing the need to design validated clinical scenarios^(23)^. However, greater investment is still needed to develop and validate scenarios for teaching a wide range of topics that may benefit from this teaching strategy in clinical skills development.

Despite the advantages of SBL for improving clinical skills and promoting the acquisition of knowledge and competencies among nursing students^(24)^, there remains a need to develop and validate simulated clinical scenarios for nursing teaching and learning across a range of topics, especially in virtual or high-fidelity formats^(8,23-26)^. Therefore, the development and validation of this scenario for teaching Standard Precautions, with an emphasis on clinical reasoning and knowledge of the steps involved in and the sequence of donning and doffing, may represent a meaningful advance for nursing education in Brazil^(23)^. Its use in undergraduate nursing programs may broaden access to knowledge and strengthen the education of future nurses. In addition, it may contribute to the training of nursing and other health professionals, including those working in remote areas.

Developing teaching scenarios requires rigorous planning and validation to ensure alignment with the learning objectives^(13,14)^. The clinical scenario developed simulated a hospital setting that requires clinical reasoning and decision-making regarding PPE selection and the recommended donning and doffing sequence. The high ratings for items such as “target audience” and “objectives” may reflect the fact that they were structured on the basis of reference models^(14)^. The robustness of the process was strengthened by combining the CVI with Gwet’s AC1 coefficient to assess agreement among the experts^(12,15)^. These findings are consistent with the literature^(8,25,26)^. However, because AC1 does not yet have a dedicated interpretation scale^(21)^ and often yields higher values than kappa, its interpretation requires caution to avoid overestimating agreement^(27)^.

Virtual simulation provides a safe and controlled environment that is essential for strengthening future nurses’ ability to adhere to Standard Precautions and use PPE appropriately. The opportunity for repetition and immediate feedback promotes learning, reduces errors, and increases student confidence, which directly supports patient and worker safety^(7,28)^. The effectiveness of simulation as a tool for improving knowledge and practice is already well documented^(28)^.

In light of these findings, health curricula should strengthen a culture of safety and infection prevention^(7,11,28)^. This study provides a validated tool for teaching Standard Precautions, while future studies should evaluate the effectiveness of such innovative technologies in the teaching and learning process.

### Study limitations

This study has some limitations. Because data collection was asynchronous and conducted online, questions could not be clarified immediately and were addressed later by email at different time points. In addition, difficulty obtaining responses from the experts extended the validation timeline. Finally, validation was limited to nursing professionals, and future studies should include participants from other health disciplines.

### Contributions to the field of nursing

This study makes a direct contribution to nursing by providing an innovative, validated educational resource focused on teaching Standard Precautions. This tool strengthens training on a critical topic, PPE use, by promoting meaningful and safe learning. The scenario is flexible and can be implemented across different educational settings: in undergraduate nursing education as a complement to the theoretical and practical components of initial training; in continuing education for nursing and other health professionals through periodic training and refresher activities; and in complementary and graduate-level courses as a tool to deepen knowledge in areas such as Infection Control and Intensive Care. By promoting an effective method for teaching and learning and for developing biosafety competencies, this study positively affects professional training, which is crucial to improving infection prevention and control practices and ensuring safe, high-quality care.

## CONCLUSIONS

The virtual simulation scenario achieved its intended purpose, showing evidence of content validity and potential suitability as a teaching tool for PPE donning and doffing. Given its innovative nature and practical applicability, validated scenarios such as this should be integrated into nursing curricula and health professional continuing education programs. Such integration is intended to foster the development of knowledge, clinical skills, and self-confidence among students and health professionals, preparing them to work in settings with a high risk of exposure to infectious agents.

## Data Availability

The research data are available in a repository: https://doi.org/10.6084/m9.figshare.28204013.v1.
