# Supplementary material for: Virtual simulation scenario for teaching standard precautions: a validation study
Source: Rev Bras Enferm. 2026 Oct 19;79:e20250346. doi: 10.1590/0034-7167-2025-0346 (PMC13679591; doi:10.1590/0034-7167-2025-0346)
Supplement: Translation

# Translation: Cenário para simulação virtual no ensino das precauções-padrão: estudo de validação

**DOI:** 10.1590/0034-7167-2025-0346pt

**Authors:** Cristine Maria Pereira Gusmão, Laís do Espirito Santo Lima, Mayra Gonçalves Menegueti, Cristina Mara Zamarioli, Elucir Gir, Renata Karina Reis

**Affiliations:** I Universidade de São Paulo. Ribeirão Preto, São Paulo, Brasil

**Keywords:** Ensino, Equipamentos de Proteção Individual, Treinamento por Simulação, Estudo de Validação, Controle de Infecção.

## Abstract

**Objetivos:**

desenvolver e validar um cenário simulado virtual para o ensino das precauções-padrão e sequência de paramentação e desparamentação dos equipamentos de proteção individual para estudantes e profissionais de enfermagem.

**Métodos:**

estudo metodológico de produção tecnológica. A construção baseou-se em recomendações científicas e diretrizes de simulação. Especialistas em simulação e controle de infecção relacionada à assistência validaram o cenário respondendo a questionários criados pelas autoras. Os dados foram analisados mediante o índice de validade de conteúdo e o coeficiente de concordância de Gwet, com valores mínimos aceitáveis de 0,80 para o IVC e 0,41 para o AC1.

**Resultados:**

validado por 11 especialistas, o cenário foi composto por 20 itens essenciais e apresentou IVC e coeficiente de Gwet superiores ao mínimo estipulado.

**Conclusões:**

o cenário validado apresenta potencial educacional e caráter inovador à simulação, contribuindo para a formação de profissionais e estudantes na prevenção de infecções.

## INTRODUÇÃO

Ambientes de assistência à saúde apresentam riscos biológicos significativos, sendo as precauções-padrão (PP) - como a higiene das mãos e o uso dos equipamentos de proteção individual (EPI) - a principal estratégia para garantir a segurança de todos^(1-3)^. Embora recomendadas por órgãos globais como a Organização Mundial da Saúde e os *Centers for Disease Control and Prevention* (CDC), a adesão às PP ainda se mostra insuficiente, tanto entre profissionais quanto entre estudantes da área da saúde^(4-6)^.

A raiz do problema está em lacunas educacionais, com currículos que não desenvolvem adequadamente competências práticas essenciais, incluindo as medidas de prevenção e controle de infecção. Achados de estudos realizados no Brasil e no exterior, somados às dificuldades observadas durante a pandemia de covid-19, revelam falhas no ensino de procedimentos cruciais, como a sequência correta de paramentação e desparamentação dos EPI^(6,7)^.

Nesse contexto, a simulação virtual surge como uma estratégia de ensino-aprendizagem eficaz, pois favorece o desenvolvimento do raciocínio clínico e de habilidades cognitivas e psicomotoras em um ambiente seguro, realista e com feedback imediato, melhorando o desempenho e a autoconfiança^(8-10)^. Contudo, seu uso no ensino das PP ainda é limitado, e faltam cenários validados para práticas específicas^(6,11)^.

## OBJETIVOS

Desenvolver e validar um cenário simulado virtual para o ensino das precauções-padrão e da sequência de paramentação e desparamentação dos equipamentos de proteção, como ferramenta pedagógica eficaz para a formação e educação continuada em enfermagem.

## MÉTODOS

### Aspectos éticos

O estudo seguiu diretrizes éticas nacionais e internacionais. Foi aprovado pelo Comitê de Ética em Pesquisa da instituição de ensino e pesquisa sob o parecer de n° 6.782.234 de abril de 2024. Todos os participantes do processo de validação foram informados sobre os objetivos do estudo e registraram eletronicamente seu consentimento no *Research Electronic Data Capture* (REDCap^®^), conforme as recomendações para pesquisas em ambiente virtual.

### Delineamento, período e local do estudo

Estudo metodológico^(12)^ de produção tecnológica realizado entre dezembro de 2023 e agosto de 2024, em uma instituição pública de ensino superior no interior do estado de São Paulo (SP). O processo seguiu o checklist *Revised Standards for Quality Improvement Reporting Excellence* (SQUIRE 2.0) para desenvolver e validar um cenário de simulação sobre precauções-padrão.

A estrutura do cenário foi baseada no *National League Nursing Jeffries Simulation Theory* - NLN/JST^(13)^ e nos padrões de boas práticas da *Healthcare Simulation Standards of Best Practice^TM^* da *International Nursing Association for Clinical Simulation and Learning* - INACSL^(14)^. Já o conteúdo técnico se fundamentou em evidências e diretrizes atuais sobre PP e uso de EPI^(1,2)^, conforme ilustrado na Figura 1.

**Figura 1:** Fluxograma do desenvolvimento do cenário virtual para o ensino de precauções-padrão, Ribeirão Preto, São Paulo, Brasil, 2025

*PP - Precauções-padrão; PA - Precauções-adicionais; EPI - Equipamento de proteção individual.

A validação de face e conteúdo foi conduzida por especialistas em simulação e/ou controle de infecção relacionada à assistência à saúde (IRAS), conforme critérios descritos na literatura^(15)^.

### População e critérios de inclusão

A seleção dos especialistas exigiu graduação na área da saúde e atendimento a um sistema de pontuação mínima adaptado de Fehring^(15)^, segundo o qual os participantes deveriam obter pelo menos cinco pontos com base em titulação acadêmica, experiência profissional e produção científica na área. A pontuação adotada foi: título de mestre na área ou tema do estudo (3 pontos); título de doutor na área ou tema do estudo (3 pontos); certificado de especialista na área do estudo (2 pontos); título de mestre/doutor em outra área (1 ponto); experiência clínica mínima de 1 ano na área de enfermagem (1 ponto); publicações em áreas como simulação e/ou controle de infecção (2 pontos); desenvolvimento de estudos na área do tema (2 pontos); orientação de trabalhos acadêmicos sobre o tema (1 ponto); experiência assistencial ou docente; e desenvolvimento de pesquisa ou autoria de artigos científicos sobre a temática em periódicos (1 ponto).

Para o recrutamento, a busca dos especialistas foi realizada na plataforma Lattes por meio da ferramenta “buscar currículo”, utilizando-se filtros por titulação, assunto e palavras-chave como “controle de infecção” e “simulação”. Em seguida, 27 profissionais foram contatados por e-mail, o qual continha as informações da pesquisa e o link para o Termo de Consentimento Livre e Esclarecido (TCLE) em formato eletrônico, com a opção de participar ou não do estudo. Com o retorno inicial de cinco especialistas, utilizou-se a técnica “bola de neve”, na qual se solicitou a indicação de profissionais com expertise no tema. Assim, foram incluídos 11 participantes - número considerado suficiente para superar o mínimo de cinco especialistas recomendado em estudos de validação^(16)^.

### Coleta de dados

Entre maio e agosto de 2024, os dados foram coletados por meio da plataforma REDCap. Após a assinatura do Termo de Consentimento Livre e Esclarecido on-line, 11 especialistas preencheram um questionário sociodemográfico e avaliaram o cenário elaborado.

A validação do cenário utilizou um questionário elaborado pelas pesquisadoras, inspirado em um estudo com proposta semelhante^(17)^. Foi composto por questões sobre o material destinadas à validação de face (clareza e realismo)^(16)^ e validação de conteúdo (pertinência e relevância)^(16)^, utilizando uma escala do tipo Likert^(18)^ com variação de 1 a 5 pontos, em que 1 correspondeu a “Não claro”, “Não pertinente” e “Não relevante” e 5 representou “Bastante claro, “Bastante pertinente” e “Bastante relevante”. Além disso, havia um campo de resposta aberta opcional para que os participantes pudessem dar sugestões para o aprimoramento do cenário.

### Análise de dados

Os resultados foram exportados do REDCap^®^ para serem processados no software R (versão 4.4.1)^(19)^, com a caracterização dos participantes feita por estatística descritiva. Na análise de concordância, empregou-se o índice de validade de conteúdo (IVC), calculado pela razão entre o número de respostas 4 e 5 e o número total de respostas dadas pelos especialistas, adotando-se como critério de aceitação valor ≥ 0,80^(12)^. Complementarmente, utilizou-se o coeficiente de concordância AC1 de Gwet (*First-order Agreement Coefficient*)^(20,21)^ para avaliar a concordância entre os especialistas. Optou-se pelo AC1 por sua robustez em comparação com as limitações do coeficiente Kappa em cenários de homogeneidade e prevalência^(20)^. A interpretação do AC1 seguiu estes critérios: < 0,40 = pobre; 0,41 a 0,75 = satisfatório a bom; 0,75 a 1,00 = excelente^(22)^. As sugestões dos participantes foram analisadas e incorporadas ao cenário para seu aprimoramento, com base na literatura.

## RESULTADOS

### Cenário

O cenário elaborado foi constituído por 20 itens. A estrutura define o nome do cenário, público-alvo, local, propósito, tempos, modalidade, nível de dificuldade, pontuação do cenário, competências prévias, referencial teórico, objetivos de aprendizagem e resultados esperados. O roteiro operacional inclui os materiais, a descrição do caso, o desenvolvimento, uma lista de verificação de ações, a avaliação e as etapas de *prebriefing* e *debriefing*, como ilustrado nas Figuras 2 e 3.

**Figura 2:** Capturas de tela do cenário virtual para o ensino de precauções-padrão validado por especialistas (N = 11), Ribeirão Preto, São Paulo, Brasil, 2025

**Figura 3:** Capturas de tela do cenário virtual para o ensino de precauções-padrão validado por especialistas (N = 11), Ribeirão Preto, São Paulo, Brasil, 2025

### Validação do cenário de simulação para o ensino de precauções-padrão

#### Caracterização dos especialistas avaliadores

A validação foi conduzida por 11 enfermeiros especialistas, sendo a maioria do sexo feminino (n = 10; 90,9%), com média de idade de 36,6 anos. O tempo médio de atuação profissional foi de 24,5 anos. O comitê de especialistas foi constituído por profissionais com pós-graduação stricto sensu, sendo seis (54,5%) doutores e cinco (45,5%) mestres.

Quanto às áreas de atuação, dez (90,9%) atuavam no ensino e na assistência; um (9,1%) tinha experiência com o uso da simulação; e um (9,1%) atuava na Enfermagem com foco no controle de infecção relacionada à assistência à saúde. A produção científica dos especialistas reforça a expertise na temática: sete (63,3%) tinham publicações sobre medidas de precaução-padrão e simulação; e oito (72,7%) sobre IRAS.

#### Evidências de validação de face e de conteúdo

Na validação de face e de conteúdo, a maioria dos itens do cenário alcançou índice de validade de conteúdo (IVC) superior a 0,80, como mostra a Tabela 1. Os itens com pontuação inferior a esse valor foram revisados com base nas sugestões dos especialistas.

**Tabela 1:** Distribuição das respostas dos especialistas quanto à clareza, pertinência e relevância do cenário de simulação para o ensino de precauções-padrão (N = 11), Ribeirão Preto, São Paulo, Brasil, 2025

Itens do cenário	Critério		
	Clareza	Pertinência	Relevância
	IVC-C^‡^	IVC-P^§^	IVC-R^||^
Nome do cenário	0,91	1,00	0,91
Público-alvo	0,91	0,91	1,00
Local para simulação e *Debriefing*	0,82	0,73	0,73
Propósito	1,00	0,91	1,00
Tempos	0,91	0,91	1,00
Modalidade	0,91	0,82	0,82
Nível de dificuldade	0,91	1,00	0,91
Pontuação do cenário	1,00	1,00	1,00
Competências prévias	0,82	0,91	1,00
Referencial teórico	0,91	0,91	0,91
Objetivos de aprendizagem	0,91	0,91	1,00
Resultados esperados	0,73	0,82	1,00
Materiais a serem selecionados	0,82	0,73	0,82
*Prebriefing*	0,91	0,91	1,00
Avaliação	0,91	1,00	1,00
Características do ambiente e roteiro	0,91	0,91	0,91
Descrição do caso	1,00	0,91	0,91
Desenvolvimento do cenário	0,91	0,91	0,91
Lista de verificação e ações esperadas	0,82	0,91	0,82
*Debriefing*	0,91	0,91	0,82

*IVC-C^‡^ - índice de validade de conteúdo quanto à clareza; IVC-P^§^ - índice de validade de conteúdo quanto à pertinência; IVC-R|| - índice de validade de conteúdo quanto à relevância.*

A concordância, avaliada pelo coeficiente AC1 de Gwet, foi considerada satisfatória entre os especialistas. Para os itens específicos do cenário, os índices foram de 0,58 para clareza, 0,47 para pertinência e 0,50 para relevância. Já para os itens gerais, os valores foram de 0,49, 0,42 e 0,53, respectivamente (Tabela 2).

**Tabela 2:** Distribuição das respostas dos especialistas quanto à concordância da validação dos itens gerais e específicos do cenário de simulação para o ensino precauções-padrão (N = 11), Ribeirão Preto, São Paulo, Brasil, 2025

	Concordância			
	**Critério**	AC1^*^	**DP^†^**	**IC^§^**
**Itens gerais do cenário** Público-alvo, local (para simulação e *debriefing*), propósito, tempos (*prebriefing, briefing* e *debriefing*), modalidade, pontuação do cenário, competências prévias, referencial teórico, objetivos de aprendizagem, resultados esperados, *prebriefing*, avaliação, materiais a serem selecionados e *debriefing*	Clareza	0,49	0,05	(0.378,0.597)
	Pertinência	0,42	0,06	(0.281,0.546)
	Relevância	0,53	0,06	(0.393,0.659)
			
				
**Itens específicos do cenário** Nome do cenário, nível de dificuldade, caracterização do ambiente e roteiro, descrição do caso, desenvolvimento do cenário, lista de verificação e ações esperadas	Clareza	0,58	0,06	(0.435,0.725)
	Pertinência	0,47	0,05	(0.353,0.588)
	Relevância	0,50	0,05	(0.373,0.616)

* *AC1 - coeficiente AC1 de Gwet First-order Agreement Coefficient; †DP - desvio-padrão; §I.C. - intervalo de confiança 95%.*

O cenário para o ensino das medidas de precaução-padrão foi aprimorado com base no retorno dos especialistas. Atendendo à solicitação de dois deles, o item “Materiais a serem selecionados” foi ajustado para “Materiais e ações a serem selecionados”.

Quanto aos itens não modificados, “Resultados esperados” e “Local para simulação e *Debriefing*” foram mantidos em sua forma original, pois não receberam sugestões específicas de mudança. Mesmo que o item “Local para simulação e *Debriefing*” tenha apresentado IVC abaixo de 0,80, a decisão de mantê-lo baseou-se em dois fatores: a ausência de propostas de alteração e a natureza do projeto. Como o cenário foi desenvolvido para uma plataforma on-line, os autores consideraram que não havia alternativas viáveis para esse tópico.

As anotações gerais no espaço para sugestões não se referiam a edições diretas no texto, mas à importância do material e à modalidade on-line da proposta, sobre a qual os participantes fizeram considerações positivas.

A versão final e consolidada do cenário, juntamente com os dados de validação, está disponível no material suplementar.

## DISCUSSÃO

Este estudo construiu e validou um cenário para simulação virtual sobre o ensino de precauções-padrão, com foco nas etapas e sequência da paramentação e desparamentação de EPI. Os resultados demonstraram evidências de validade de face e de conteúdo para o contexto proposto, com índices de validade de conteúdo acima de 0,80 e um coeficiente de concordância satisfatório entre os especialistas.

No Brasil, é crescente o uso da aprendizagem baseada em simulação (ABS) pela enfermagem na contemporaneidade, o que desafia os educadores e exige, cada vez mais, o planejamento de design de cenários clínicos validados^(23)^. No entanto, ainda há necessidade de maior investimento na construção e validação de cenários para o ensino de diferentes temáticas, que podem se beneficiar dessa estratégia pedagógica no desenvolvimento de habilidades clínicas.

Apesar das vantagens da ABS para o aprimoramento das habilidades clínicas e da aquisição de conhecimento e competências entre estudantes de Enfermagem^(24)^, o planejamento de design de cenários clínicos simulados e validados para o ensino-aprendizagem em enfermagem em diversas temáticas é necessário especialmente em formatos virtuais ou de alta fidelidade^(8,23-26)^. Portanto, o desenvolvimento e validação desse cenário para o ensino das PP, com enfoque no raciocínio clínico e conhecimento das etapas e da sequência da paramentação e desparamentação, podem representar um avanço para as instituições formadoras brasileiras^(23)^. Sua utilização nos cursos de graduação em Enfermagem pode contribuir para democratizar o conhecimento e fortalecer a formação dos futuros enfermeiros. Além disso, pode colaborar para a qualificação de profissionais de enfermagem e saúde, incluindo aqueles que atuam em áreas remotas.

A construção de cenários de ensino exige planejamento e validação rigorosos para garantir o alinhamento aos objetivos de aprendizagem^(13,14)^. O cenário clínico elaborado reproduziu um ambiente hospitalar, que exige raciocínio clínico e tomada de decisão quanto à seleção dos EPI na sequência recomendada. A alta pontuação de itens como “público-alvo” e “objetivos” pode ser atribuída à sua estruturação baseada em modelos de referência^(14)^. A robustez do processo foi reforçada pela combinação do IVC com o coeficiente AC1, que mediu a concordância entre os especialistas^(12,15)^. Esses achados corroboram a literatura^(8,25,26)^. Contudo, como o AC1 ainda carece de uma escala de interpretação própria^(21)^ e frequentemente produz valores mais altos que o Kappa, sua interpretação exige cautela para evitar a superestimação da concordância^(27)^.

A simulação virtual oferece um ambiente seguro e controlado, essencial para fortalecer a capacidade dos futuros profissionais de enfermagem de aderir às precauções-padrão e usar adequadamente os EPI. A possibilidade de repetição e do feedback imediato favorece o aprendizado, a redução de erros e o aumento da confiança do estudante, o que impacta diretamente a segurança do paciente e do profissional^(7,28)^. A eficácia da simulação como ferramenta para melhorar o conhecimento e a prática já é bem documentada^(28)^.

Diante disso, recomenda-se que os currículos da área da saúde reforcem uma cultura de segurança e prevenção de infecções^(7,11,28)^. Este estudo oferece uma ferramenta validada para o ensino das PP, ao passo que estudos posteriores devem avaliar a efetividade de tais tecnologias inovadoras no processo de ensino-aprendizagem.

### Limitações do estudo

O estudo apresenta algumas limitações. A coleta de dados, por ser assíncrona e virtual, impediu o esclarecimento imediato de dúvidas, que foram tratadas posteriormente por e-mail, em momentos distintos. Além disso, a dificuldade em obter o retorno dos especialistas prolongou o cronograma da validação. Por fim, a validação foi restrita a profissionais de enfermagem, sendo recomendável a inclusão de outras áreas da saúde em pesquisas futuras.

### Contribuições para a área da enfermagem

Este estudo contribui diretamente para a enfermagem ao disponibilizar um recurso educacional inovador e validado, focado no ensino de precauções-padrão. A ferramenta fortalece a capacitação em um tema crítico (o uso de EPI), promovendo uma aprendizagem significativa e segura. O cenário é aplicável e flexível, podendo ser implementado em diferentes contextos educacionais, tais como no ensino de graduação em Enfermagem enquanto método complementar à teoria e à prática da formação inicial; na educação permanente de profissionais de enfermagem e de saúde, por meio de capacitações periódicas e ações de atualização; em cursos complementares e de pós graduação na qualidade de ferramenta complementar ao aprofundamento do conhecimento em áreas como Controle de Infecção e Terapia Intensiva. Ao promover um método eficaz para o ensino aprendizagem e desenvolvimento de competências em biossegurança, a pesquisa impacta positivamente a formação profissional, o que é crucial para melhorar as práticas de controle de infecção e assegurar uma assistência qualificada e segura.

## CONCLUSÕES

O cenário simulado virtual desenvolvido atingiu o objetivo proposto, apresentando evidências de validade de conteúdo e adequação potencial como ferramenta de ensino para as etapas de paramentação e desparamentação de EPI. Diante de seu caráter inovador e aplicabilidade prática, recomenda-se a incorporação de cenários validados como este aos currículos acadêmicos de Enfermagem e à educação permanente de profissionais da área da saúde. Tal integração visa promover o desenvolvimento de conhecimento, habilidades clínicas e autoconfiança nos estudantes e profissionais de saúde, preparando-os para atuar em ambientes com alto risco de exposição a agentes infecciosos.

## Data Availability

Os dados de pesquisa estão disponíveis em repositório: https://doi.org/10.6084/m9.figshare.28204013.v1.
